# 5 V Compatible Two-Axis PZT Driven MEMS Scanning Mirror with Mechanical Leverage Structure for Miniature LiDAR Application

**DOI:** 10.3390/s17030521

**Published:** 2017-03-05

**Authors:** Liangchen Ye, Gaofei Zhang, Zheng You

**Affiliations:** State Key Laboratory of Precision Measurement Technology and Instruments, Department of Precision Instrument, Tsinghua University, Beijing 100084, China; ylc12@mails.tsinghua.edu.cn

**Keywords:** 5 V compatible, MEMS scanning mirror, piezoelectric, PZT ceramic, mechanical leverage, impedance matching

## Abstract

The MEMS (Micro-Electronical Mechanical System) scanning mirror is an optical MEMS device that can scan laser beams across one or two dimensions. MEMS scanning mirrors can be applied in a variety of applications, such as laser display, bio-medical imaging and Light Detection and Ranging (LiDAR). These commercial applications have recently created a great demand for low-driving-voltage and low-power MEMS mirrors. However, no reported two-axis MEMS scanning mirror is available for usage in a universal supplying voltage such as 5 V. In this paper, we present an ultra-low voltage driven two-axis MEMS scanning mirror which is 5 V compatible. In order to realize low voltage and low power, a two-axis MEMS scanning mirror with mechanical leverage driven by PZT (Lead zirconate titanate) ceramic is designed, modeled, fabricated and characterized. To further decrease the power of the MEMS scanning mirror, a new method of impedance matching for PZT ceramic driven by a two-frequency mixed signal is established. As experimental results show, this MEMS scanning mirror reaches a two-axis scanning angle of 41.9° × 40.3° at a total driving voltage of 4.2 Vpp and total power of 16 mW. The effective diameter of reflection of the mirror is 2 mm and the operating frequencies of two-axis scanning are 947.51 Hz and 1464.66 Hz, respectively.

## 1. Introduction

MEMS scanning mirrors have been used in many applications including confocal microscopy [1,2], biomedical imaging [3,4], head-up displays [5] and Light Detection and Ranging (LiDAR) [6,7,8,9]. Nowadays, these applications, especially LiDAR for unmanned driving or unmanned aerial vehicle (UAV), have recently created a great demand for low-cost, low-dissipation and low-weighted two-axis MEMS scanning mirrors. With the development of MEMS (Micro-Electro-Mechanical System) technology, the MEMS scanning mirror’s advantages make it probably the most suitable laser beam scanner for LiDAR. The MEMS scanner has the advantages of high operating frequency, low-weight and small volume of package. However, most MEMS scanners have a high operating voltage or high power which makes them incapable of being universally integrated in LiDAR.

Efforts have been made to decrease MEMS scanning mirrors’ driving voltage and power consumption. A variety of actuations and driving structures have been demonstrated. Electrostatic actuators have the characteristic of extremely high driving voltage but low power. Although using the wafer-level vacuum packaging technology, a total driving voltage of 70 V is still needed to actuate a two-axis MEMS scanning mirror for an electrical MEMS scanner with a FOV (Field of view) of 60° and 70° respectively [10]. Moreover, the power of a high-voltage driving circuit is much higher than the power of the mirror. Electromagnetic MEMS scanning mirrors are excited by current and have a low driving voltage. However, this kind of actuator has higher power consumption, for example, one hundred to some hundreds milliwatts [11,12]. 

Piezoelectric actuators have the advantage of yielding a high force with a smaller driving voltage compared to other driving actuators. Bulk PZTs are especially suitable for the MEMS scanning mirror for a simple fabrication process and fast response. Chen [13] presented a MEMS scanning mirror with a Y-shaped piezoelectric actuator for projector application. The power consumption of the scanner is 13.4 mW at a driving voltage of 10 Vpp in each axis, while the FOV is 27.6° and 39.9° respectively. The operating frequencies of two-axis scanning are 560 Hz and 25 kHz, but the diameter of the mirror plate is 1 mm which is too small to reflect the measuring laser beam in long-range LiDAR applications [14]. The MEMS scanning mirror with PZT has the potential for lower voltage and power consumption. However, no 5 V compatible 2D MEMS scanning mirror with a large FOV has been reported yet. Five volts is the most universal voltage in current commercial systems. The boost DC/DC converter is no longer needed with a 5 V compatible MEMS scanning mirror. Furthermore, high-voltage circuits for driving will occupy more static power in circuit than low-voltage circuits because the static driving current of the operational amplifier (OP) goes higher when the driving voltage is higher.

In this work, we present a new piezoelectric MEMS scanning mirror with mechanical leverage to decrease the driving voltage and we obtained a 5 V compatible MEMS scanning mirror with a large FOV. An impedance matching method of PZT ceramic is established to decrease the reactive power of PZT ceramic. As the results show, in order to actuate a two-axis MEMS scan mirror with scanning angles of 41.9° × 40.3°, a total voltage of 4.2 Vpp and total power of 16 mW are achieved.

## 2. Design and Model of Two-Axis MEMS Scanner

**Figure 1:**
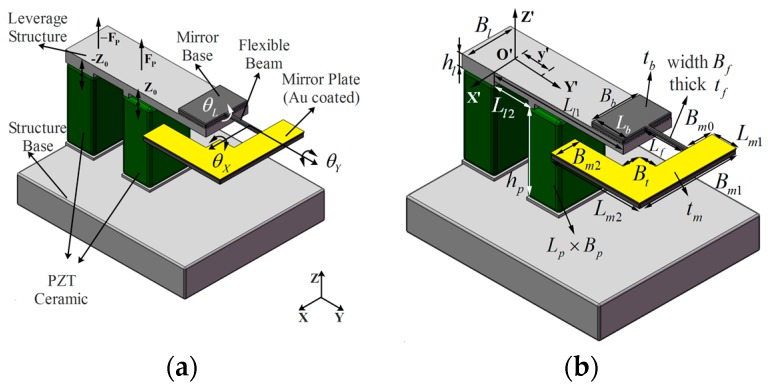
(**a**) A sketch of the Micro-Electronical Mechanical System (MEMS) scanning mirror with two Lead zirconate titanate (PZT) ceramics; (**b**) Parameters definitions of the device.

Figure 1 shows the sketch of the MEMS scanning mirror. The device comprises a PZT ceramic actuator, a leverage structure and a Si-based MEMS structure. The MEMS scanning mirror consists of a mirror base, a flexible beam and a mirror plate which is coated with Au film to increase the reflective coefficient of the reflector. The devices (see Figure 1a) contain two PZT ceramics to which a driving voltage is applied with a 180° phase lag with each other. The PZT ceramic can realize a *Z*-axis vibration at one end of the ceramic when applying an AC driving voltage. The amplitude of PZT ceramic’s vibration is small (about some micrometers) while the output force (some hundred Newtons) is too large to actuate this MEMS device, which has a small moment of inertia. To improve the efficiency of the PZT actuator, the leverage structure is utilized to magnify the amplitude of the PZT ceramic. A Si-based MEMS structure is attached at the end of leverage and the displacement amplitude of the mirror base is increased many more times than the output amplitude of PZT ceramic itself. Two modes of the MEMS scanning mirror’s vibration are used to scan the laser beam. The first mode is the twisting mode in which the mirror plate rotates along the flexible beam (*Y*-axis). The bending mode is another scan type while the rotational axis is parallel to the *X*-axis.

**Figure 2:**
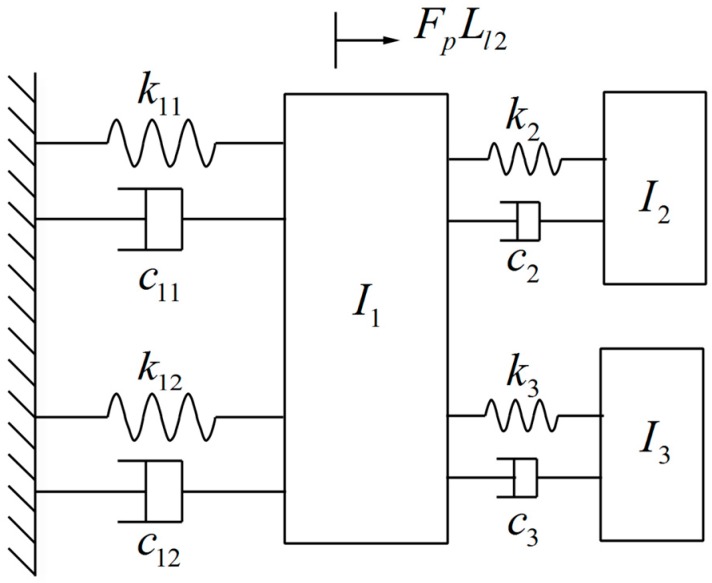
Model of the 3-DOF two-axis MEMS scanning mirror.

Figure 2 shows the three DOF (degree of freedom) vibrating model for the MEMS mirror. The terms $\theta_{L}$, $\theta_{X}$ and $\theta_{Y}$ in Figure 1a are the rotational displacement of the three sub-structures. The first sub-structure contains two PZT ceramics and a leverage: $I_{1}$ is the equivalent moment of inertia of the first sub-structure, $k_{11}$ and $c_{11}$ are the stiffness and damping coefficients of the two PZT ceramic system, and $k_{12}$ and $c_{12}$ are the stiffness and damping coefficients of the leverage. In this model, only the twisting and bending mode of the MEMS mirror are considered. The second sub-structure contains the flexible beam and mirror plate: $\theta_{Y}$, $k_{2}$, $c_{2}$ and $I_{2}$ are the mechanical angle, torsional stiffness, damping coefficient and mass moment of inertia of the twisting-mode rotational model. The third sub-structure contains the flexible beam and mirror plate: $\theta_{X}$, $k_{3}$, $c_{3}$ and $I_{3}$ are the mechanical angle, bending stiffness, damping coefficient and mass moment of inertia of the bending-mode rotational model. The flexible beam can twist and bend at the same time. 

The force generated by PZT ceramic can be estimated as:

(1) $$F_{P}=nd_{33}E_{P}\frac{A_{P}}{h_{P}}U$$

where *n* is the number of the element in the PZT ceramic, U is the applied voltage, and $d_{33}$, $E_{P}$, $A_{P}$, $h_{P}$ are the piezoelectric constant, elastic module, sectional area and height of the PZT ceramic.

**Figure 3:**
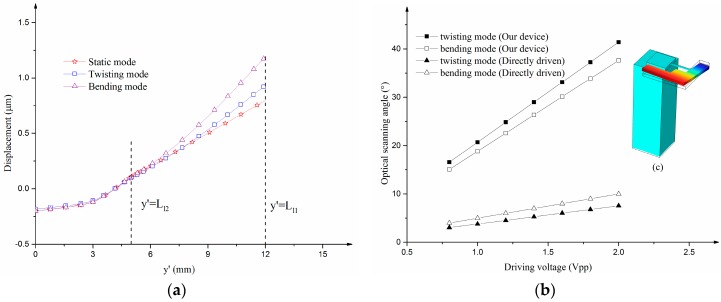
(**a**) *Z*-axis displacement at different positions on the bending leverage (the middle PZT is at the position of $y^{\prime }=L_{l2}$ and the end of the leverage is at the position of $y^{\prime }=L_{l1}$); (**b**) The optical scanning angle response of the device in comparison with the device directly driven by PZT.

FEM (Finite element method) simulation is applied to solve the complex model of the MEMS scanning mirror. The parameters of the device are listed in Table 1. Figure 3a shows the *Z*-axis displacement at different positions on the bending leverage. The leverage structure can magnify the amplitude of the PZT ceramic. The parameter $y^{\prime }$ defines the distance between the point on the leverage and the pivot (*O’*) along the *Y’*-axis. The amplitude of displacement at the end of the leverage ($y^{\prime }=L_{l1}$) is much larger than the amplitude of the PZT ceramic. Figure 3b shows the simulated optical scanning angle of the twisting mode and bending mode as a function of driving voltage. The optical scanning angle of the device is four to five times larger than the MEMS scanning mirror directly driven by the PZT ceramic (see Figure 3c). From the curve of the single directly driven scanner, the optical scanning angle becomes larger as the driving voltage increases which is proportional to the amplitude of displacement at the mirror base. Leverage’s function of amplifying the amplitude of displacement leads to the decrease in driving voltage. Much lower driving voltage is needed to achieve the same scanning angles. 

**Figure 4:**
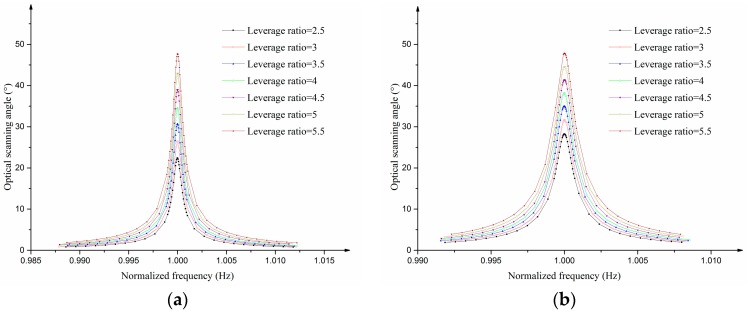

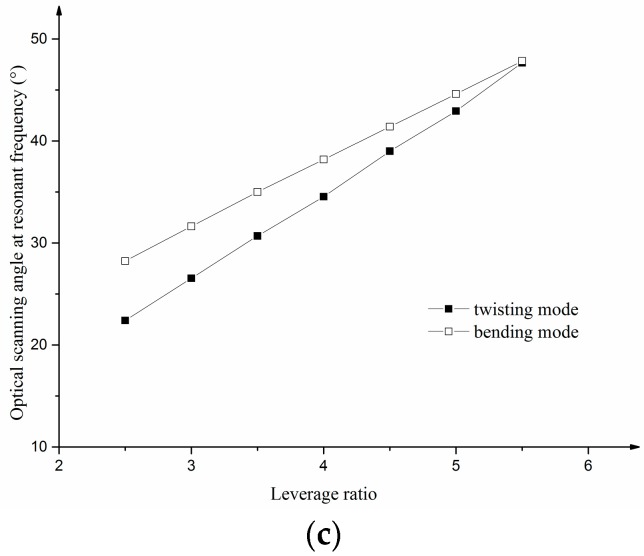
Analysis results from FEM simulation: (**a**) Frequency response of the optical scanning angle in the twisting mode; (**b**) Frequency response of the optical scanning angle in the bending mode; (**c**) The relationship between the optical scanning angle at resonant frequency and the leverage ratio.

Figure 4a,b shows the frequency response of the optical scanning angle in the twisting and bending mode. The normalized frequency in these two figures can be calculated by dividing the frequency by the resonant frequency. In these simulations, the damping ratios of the twisting mode and bending mode are set as 0.00042 and 0.0005 which can be achieved by frequency–domain FSI (fluid–solid interaction) simulation. The FSI simulation can be conducted using frequency–domain linearized Navier–Stoke and the Solid Mechanics module in COMSOL Multiphysics. The optical scanning angles at the two resonant frequencies increase as the leverage ratio increases (see Figure 4c) and the leverage ratio can be estimated as:

(2) $$k_{r}=\frac{L_{l1}}{L_{l2}}$$

where $L_{l1}$ is the distance between the pivot and the end of the leverage and $L_{l2}$ is the distance between the pivot and the attachment between the middle PZT ceramic and the leverage.

**Figure 5:**
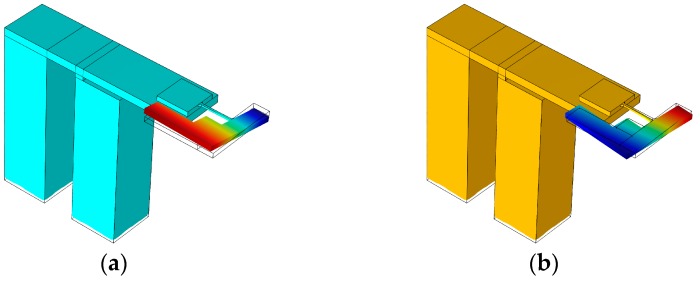

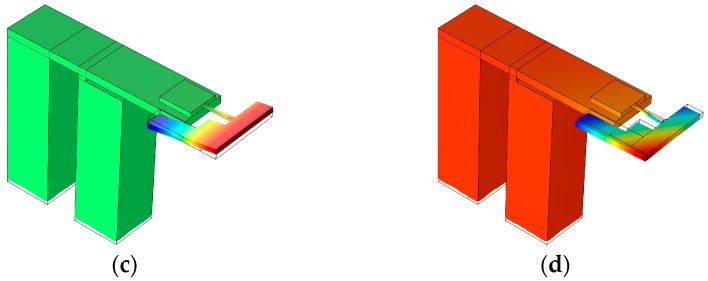
Modal analysis results from finite-element simulation. (**a**) Twisting mode; (**b**) In-plane vibration model; (**c**) Bending mode; (**d**) Shifting model.

A finite-element modal analysis is performed; the results of the MEMS scanning mirror are shown in Figure 5. The first modal frequency is 997.2 Hz (see Figure 5a) at which the scanning mirror vibrates along the flexible beam (called the twisting mode). The second modal is in-plain vibration (see Figure 5b). At the third modal frequency of 1408.4 Hz (see Figure 5c), the scanner rotates along the *X*-axis (called the bending mode). The fourth modal is much higher than the frequency of bending mode vibration at which the mirror shifts along the *Z*-axis (see Figure 5d). Two vibration modes are utilized for scanning in our device: the first mode (or twisting mode) and third mode (or bending mode). When applying a two-frequency mixed driving signal between the two electrodes of the PZT ceramic, the reflected mirror rotates along the flexible beam and *X*-axis at the same time to achieve a two-axis scan.

## 3. Impedance Matching for Power Supply of MEMS Scanner

There are some other problems when applying PZT ceramics in the MEMS scanning mirror. The power of this actuator is high in some applications in relation to its large capacity which will result in high wattless power. Impedance matching of the ultrasonic transducer working in ultrasonic resonant frequency has been investigated [15]. In this chapter, impedance matching for PZT ceramic operating in two low mixed frequencies is modeled for a two-axis MEMS scanner.

**Figure 6:**
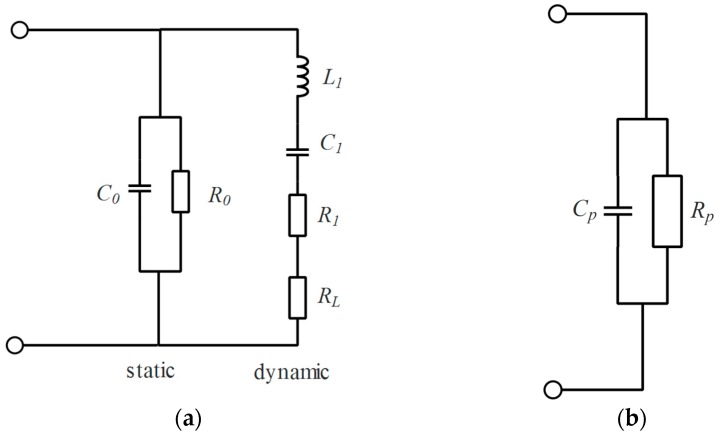

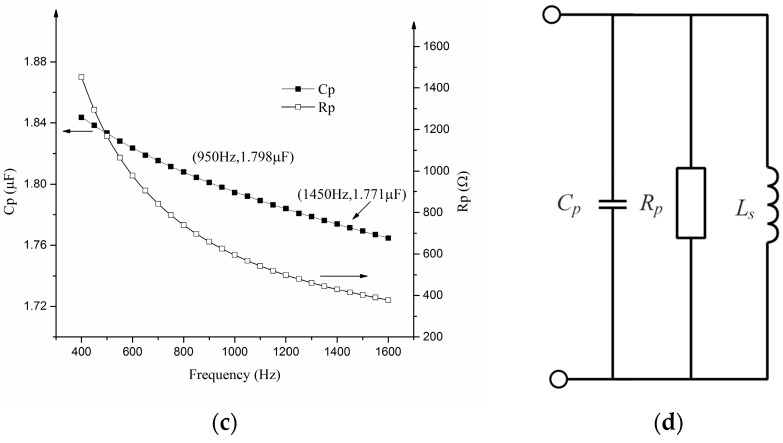
(**a**) Mason’s equivalent circuit of PZT ceramic; (**b**) Simplified equivalent circuit of PZT ceramic; (**c**) Measured parallel capacity and resistor at different frequencies; (**d**) Schematic diagram of the impedance matching method.

The circuit model of PZT ceramic can be established using Mason’s Equivalent circuit (see Figure 6a). In this model, $C_{0}$ represents the capacitance of the piezoelectric material and represents dielectric losses within the ceramics and can be neglected. The dynamic vibration of PZT ceramic is described by dynamic inductor ($L_{1}$), dynamic capacity ($C_{1}$) and mechanical dissipation resistance ($R_{1}$). Acoustic radiation of PZT ceramic is represented by resistance ($R_{L}$). 

The model of PZT ceramic can be simplified by the “$C_{P}-R_{P}$” model, as shown in Figure 6b. The parallel capacity ($C_{P}$) and parallel resistance ($R_{P}$) can be calculated by solving the equation:

(3) $$\frac{1}{Z}=\frac{1}{1/(i\omega C_{0})}+\frac{1}{i\omega L_{1}+1/(i\omega C_{1})+R_{1}+R_{L}}=\frac{1}{1/(i\omega C_{P})}+\frac{1}{R_{P}}$$

where Z is the impedance of the circuit in Figure 6a.

We measured the impedance of PZT ceramic using a precision impedance analyzer (Agilent E4980, Agilent Technologies, Santa Clara, CA, USA). As the impedance results show (see Figure 6c), the parallel capacity ($C_{P}$) of PZT ceramic only changes about 1.5% from 950 Hz to 1450 Hz. The change of parallel resistance ($R_{P}$) is much larger and much more sensitive to frequency because mechanical vibration-based mechanical dissipation resistance ($R_{1}$) changes a lot as the frequency changes.

PZT ceramic of NAC2002-H12 is used in our MEMS scanning mirror. This PZT ceramic has about 1.8 micro-farads capacity (impedance of 88.4 ohms at a frequency of 1 kHz) and some hundreds ohms parallel resistor. Reactive power is more than 25 times larger than valid power. To acquire more valid power consumption from the power supplement, impedance matching must be considered. The goal is to lower the reactive power consumption which is caused by parallel capacity ($C_{P}$). A matching parallel inductor ($L_{s}$) is introduced in parallel with PZT ceramic (see Figure 6d).

In this application, two mixed frequency excitations are applied and the amplitude of the two excitations is $U_{1}$ and $U_{2}$ while the frequency is $f_{1}$ and$f_{2}$. The total power consumption before impedance matching is:

(4) $$S=U_{eff}I_{eff}=\frac{1}{2}\sqrt{U_{1}^{2}+U_{2}^{2}}\sqrt{(2\pi U_{1}f_{1}C_{P}{)}^{2}+\frac{U_{1}^{2}}{R_{P1}^{2}}+(2\pi U_{2}f_{2}C_{P}{)}^{2}+\frac{U_{2}^{2}}{R_{P2}^{2}}}$$

where $R_{P1}$ and $R_{P2}$ are parallel resistances at $f_{1}$ and $f_{2}$ respectively.

The total power consumption after compensation can be calculated as:

(5) $$S^{\prime }=U_{eff}^{\prime }I_{eff}^{\prime }=\frac{1}{2}\sqrt{U_{1}^{2}+U_{2}^{2}}\sqrt{(2\pi U_{1}f_{1}C_{P}-\frac{U_{1}}{2\pi f_{1}L_{s}}{)}^{2}+\frac{U_{1}^{2}}{R_{P1}^{2}}+(2\pi U_{2}f_{2}C_{P}-\frac{U_{2}}{2\pi f_{2}L_{s}}{)}^{2}+\frac{U_{2}^{2}}{R_{P2}^{2}}}$$

The best optimization inductance of the parallel inductor ($L_{C}$) can be generated by calculating the minimum value of Equation (4). To find the local extremum, we take one differential of the above equation (∂S′/∂L) and set this differential equation to zero (see Equation (6)).

(6) $$\frac{\partial S^{\prime }}{\partial L_{0}}=\frac{\sqrt{U_{1}^{2}+U_{2}^{2}}[(2\pi U_{1}f_{1}C_{P}-\frac{U_{1}}{2\pi f_{1}L_{s}})\frac{U_{1}}{f_{1}}+(2\pi U_{2}f_{2}C_{p}-\frac{U_{2}}{2\pi f_{2}L_{s}})\frac{U_{2}}{f_{2}}]}{4\pi L_{0}^{2}\sqrt{(2\pi U_{1}f_{1}C_{P}-\frac{U_{1}}{2\pi f_{1}L_{s}}{)}^{2}+\frac{U_{1}^{2}}{R_{P1}^{2}}+(2\pi U_{2}f_{2}C_{0}-\frac{U_{2}}{2\pi f_{2}L_{s}}{)}^{2}+\frac{U_{2}^{2}}{R_{P2}^{2}}}}=0$$

We can achieve the optimal inductance by solving the above equation. The optimal inductance when minimum power consumption can be achieved is:

(7) $$L_{C}=\frac{U_{1}^{2}/f_{1}^{2}+U_{2}^{2}/f_{2}^{2}}{4\pi^{2}C_{P}(U_{1}^{2}+U_{2}^{2})}$$

**Figure 7:**
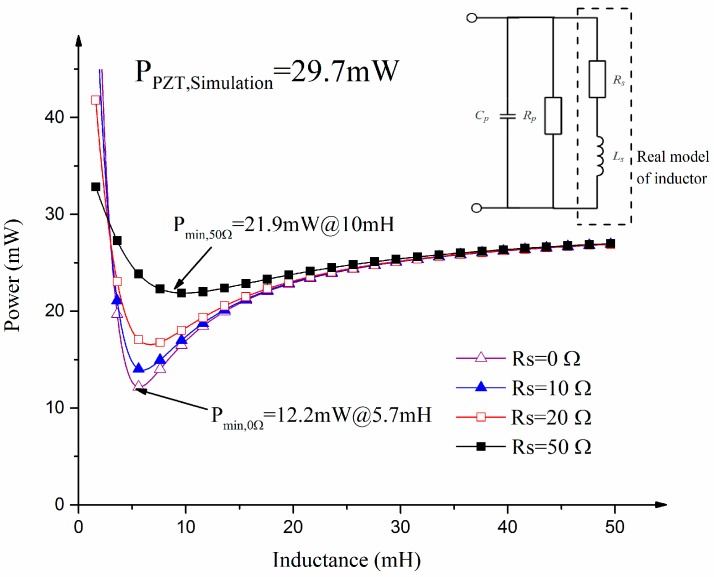
Variable simulated power consumptions versus compensational inductance.

In fact, inductors are not ideal components and have internal resistance. Their circuit model contains an inductor and a series-wound resistor (see Figure 7). Figure 7 illustrates the simulated power consumption of the impedance matching circuit with a variable series-wound resistor. The power consumption of the PZT ceramic alone is 29.7 mW. The minimum power is 12.2 mW, 13.9 mW, 16.5 mW and 21.9 mW when the series-wound resistor ($R_{s}$) is 0 Ω, 10 Ω, 20 Ω, and 50 Ω respectively. In comparison with the power of the MEMS scanning mirror without impedance matching, the power with impedance matching can be reduced to 12.2 mW which is about three times less than PZT ceramic alone.

## 4. Fabrication and Assemble

**Figure 8:**
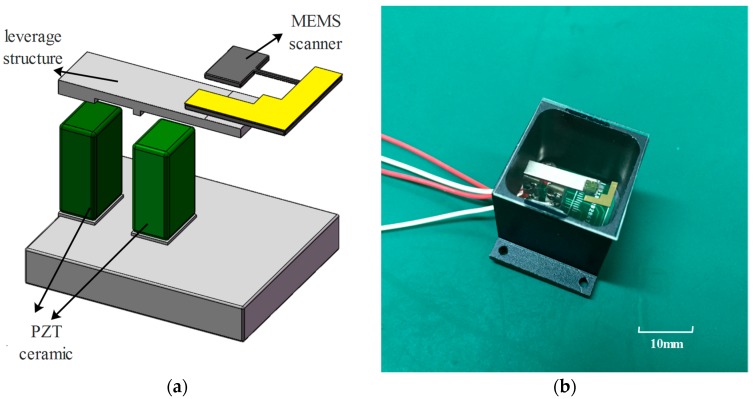
(**a**) Assembly of the MEMS scanning mirror and (**b**) Package of the MEMS scanning mirror.

The MEMS scanning mirror was produced using the bulk MEMS fabrication techniques. A piezoelectric angle position sensor is integrated on the flexible beam on the Si-based structure to measure the angle position of both axis [16]. Two PZT ceramics, a circuit board and a Titanium alloy beam are glued using 3M instant adhesive glue CA40H (Minnesota Mining and Manufacturing Company, St. Paul, MN, USA) to form the base of the MEMS scanner (see Figure 8a). The Si-based mirror is then glued to the actuator using CA40H. The entire structure is assembled into an Aluminum-alloy package with a glass window on it to protect the structure from damage and disruption of airflow. Driving voltage is applied to the drive line on the PZT ceramic. Figure 8b shows the completed assembly of the scanning device.

## 5. Measurement

In this chapter, features of the MEMS scanning mirror were experimented. Resonant Frequency and optical scan angle were the key characteristics of the MEMS scanning mirror. These two parameters were measured in this section. Method of impedance matching was also realized in this chapter and the power of the MEMS scanning mirror was measured.

### 5.1. Frequency and Optical Scanning Angle Test

**Figure 9:**
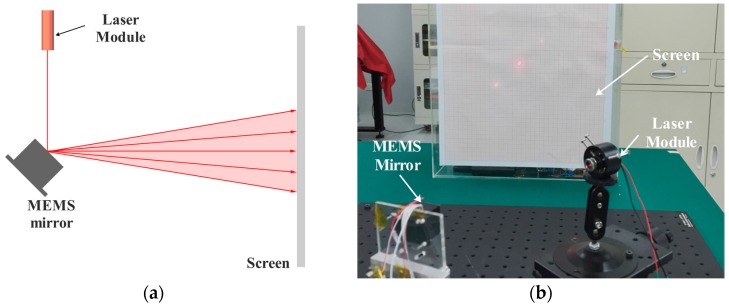
(**a**) Diagram of the experimental setup; (**b**) The experimental setup.

Figure 9 shows the experimental setup for measuring the scanning angles of the MEMS scanning mirror. A red laser emitted towards the MEMS scanning mirror was reflected by the resonant mirror and then the laser beam was reflected to the screen. The optical scanning angle could be calculated from the length of the laser line on the screen and the distance between the MEMS scanning mirror and the screen. The driving voltage applied between two poles of the PZT ceramic was a mixture frequency signal and the frequencies of two signal were $f_{1}$ and $f_{2}$ respectively. The driving voltage was generated by a data acquisition card (NI USB-6216, National Instruments, Austin, TX, USA) and then amplified by an operational amplifier (OP).

**Figure 10:**
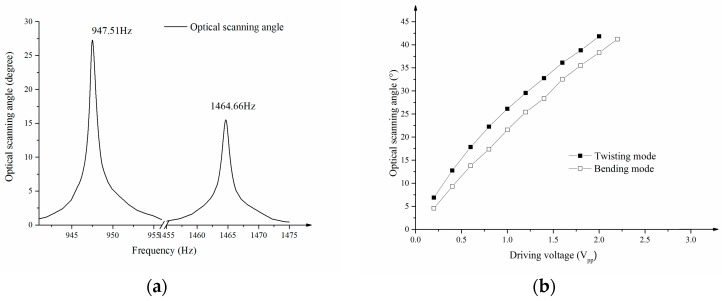
(**a**) Frequency spectrum of the optical scanning angles of the MEMS scanning mirror; (**b**) the optical scanning angles versus the applied variable voltage in the two rotation axes.

Figure 10a illustrates the relationship between the optical scanning angles and driving frequency measured by Laser Doppler Velocimetry (LDV). The amplitude of vibration at the margin of the mirror plate was transmitted to the scanning angle using the geometrical relationship. The driving frequencies of the two rotational axes were 947.51 Hz and 1464.66 Hz and the Q values were 1289 and 1046 respectively. The frequency band widths of the two-axis MEMS scanning mirror were so narrow that the cross-coupling between the twisting and bending mode could be neglected. Figure 10b shows the relationship between the optical scanning angle and driving voltage in the two rotational axes. As introduced in chapter 2, the device contained two PZT ceramics to which two different driving voltages were applied with a 180° phase lag with each other. When measuring the optical scanning angle of one rotational axis, the driving voltage at the resonant frequency of this mode was applied. The twisting mode of the device was able to achieve a 41.9° optical angle with a driving voltage of 2 Vpp. To achieve a 41.2° optical angle in the bending mode, the driving voltage was 2.2 Vpp.

**Figure 11:**
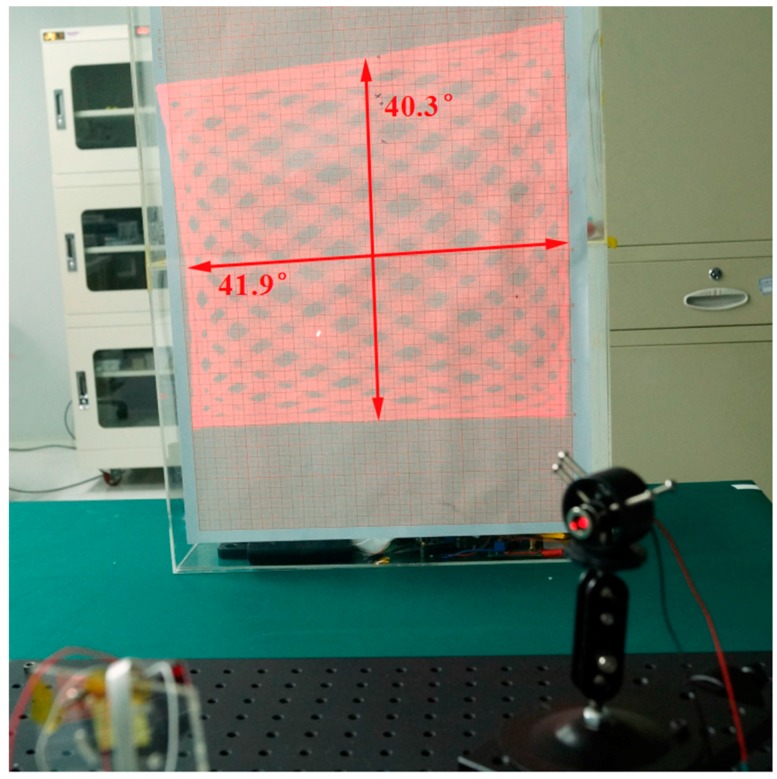
A two-dimensional pattern scanned by the MEMS scanning mirror.

When driving the device using a two-frequency mixed signal, the MEMS scanning mirror could project a two-dimensional pattern (see Figure 11). The driving frequencies of the two rotational axes were 947.51 Hz and 1464.66 Hz, and the optical scanning angles were 41.9° and 40.3°. It was unavoidable that the rotation of the two axes affect each other and a decrease in the optical scanning angle was observed in comparison with the result in Figure 10b.

### 5.2. Results of Impedance Matching

**Figure 12:**
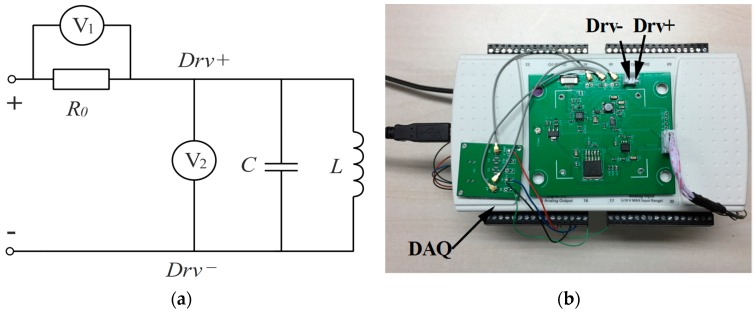
(**a**) Diagram of the measuring power circuit system; (**b**) Hardware devices of the measurement system.

The efficiency of impedance matching was validated in this section. A power measurement circuit (see Figure 12a) was applied to measure the power of the MEMS scanning mirror alone or with the impedance matching circuit. The driving current was measured by measuring the voltage of a sample resistor when the device operated at its resonant frequency. The driving voltage and current were monitored by a data acquisition device (NI USB-6216) and the power consumption was calculated by multiplying the effective value of the voltage and the effective value of the current in a LabVIEW programme according to Equation (4) (see Figure 12b).

**Figure 13:**
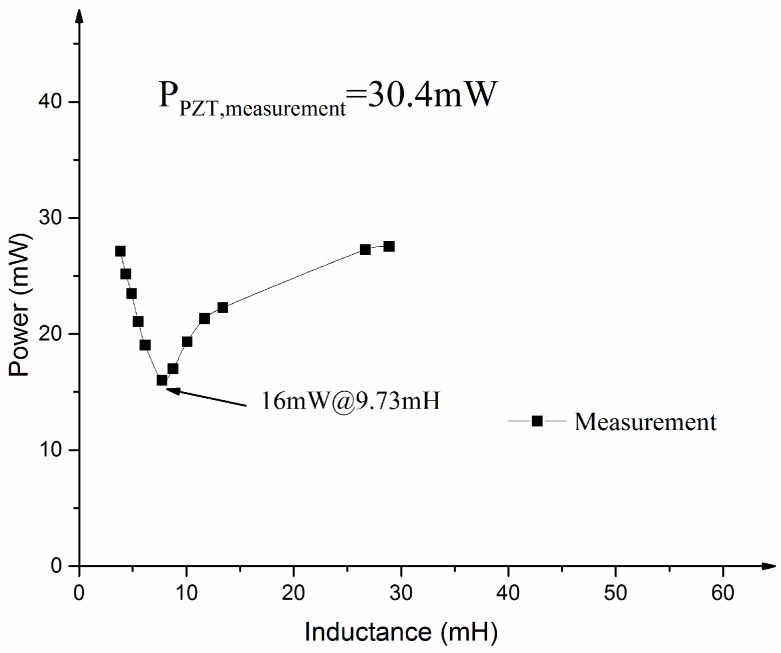
Power of the MEMS scanning mirror with the impedance matching circuit in comparison with simulation results.

Inductors with variable values were applied to the impedance matching circuit of the MEMS scanning mirror. The above measuring system was used to measure the power of the MEMS scanner with or without the impedance matching circuit. The driving voltage of the MEMS scanning mirror was 2 Vpp at 947.51 Hz, increased by 2.2 Vpp at 1464.66 Hz. The minimum power of the MEMS scanning mirror with the impedance matching circuit was 16 mW while it was 30.4 mW without the impedance matching circuit (see Figure 13). A total of 47.4% power was saved using the impedance matching method.

## 6. Conclusions

A 5 V compatible two-axis MEMS scanning mirror with a large FOV is presented in this paper for the first time. The mechanical leverage was designed to decrease the driving voltage of the MEMS scanning mirror. An impedance matching method for PZT ceramic driven by a two-frequency mixed signal was built to decrease the reactive power and total power of the MEMS scanning mirror. Full optical scanning angles of 41.9° and 40.3° were achieved at a total voltage of 4.2 Vpp for the twisting axis and bending axis respectively. The driving frequencies of the two rotational axes were 947.51 Hz and 1464.66 Hz with a mirror size of 2 mm. The power consumption of the MEMS scanning mirror can be decreased to 16 mW when applying the method of impedance matching. The 5 V compatible MEMS scanning mirror with very low power consumption can broaden the usage of the MEMS scanning mirror in miniature applications. Future work will focus on its application in LiDAR systems.

## Figures and Tables

**Table 1:** Parameters of the MEMS scanning mirror.

Parameter	Value	Parameter	Value
$L_{l1}$	9 mm	$L_{m1}$	1 mm
$L_{l2}$	2 mm	$L_{m2}$	4.35 mm
$B_{l}$	3 mm	$B_{t}$	1 mm
$h_{l}$	0.5 mm	$B_{m0}$	1.85 mm
$L_{b}$	2 mm	$B_{m1}$	4.62 mm
$B_{b}$	2 mm	$B_{m2}$	1 mm
$t_{b}$	0.3 mm	$t_{m}$	0.3 mm
$L_{f}$	2 mm	$L_{P}$	3 mm
$B_{f}$	0.12 mm	$B_{P}$	3 mm
$t_{f}$	0.105 mm	$h_{P}$	10 mm
